# Is there an association between molar incisor hypomineralization and developmental dental anomalies? A case-control study

**DOI:** 10.1186/s12903-023-03540-8

**Published:** 2023-10-21

**Authors:** Betül Şen Yavuz, Berkant Sezer, Remziye Kaya, Nihan Tuğcu, Betül Kargül

**Affiliations:** 1 Department of Pediatric Dentistry, School of Dentistry, Marmara University, Istanbul, Türkiye; 2 Department of Pediatric Dentistry, School of Dentistry, Çanakkale Onsekiz Mart University, Çanakkale, Türkiye; 3 Private Practice, Istanbul, Türkiye

**Keywords:** Developmental dental anomalies, Molar incisor hypomineralization, Panoramic radiograph

## Abstract

**Background:**

The aim of this study was to determine whether there is any association between molar incisor hypomineralization and developmental dental anomalies.

**Methods:**

Two pediatric dentists evaluated panoramic radiographs of 429 children aged 8–14 years with molar incisor hypomineralization (study group) and 437 children without molar incisor hypomineralization (control group) in terms of developmental dental anomalies. Twelve different developmental dental anomalies were categorized into four types: size (microdontia, macrodontia); position (ectopic eruption of maxillary permanent first molars, infraocclusion of primary molars); shape (fusion, gemination, dilaceration, taurodontism, peg-shaped maxillary lateral incisors); and number (hypodontia, oligodontia, hyperdontia) anomalies.

**Results:**

No significant difference was observed in the frequencies of developmental dental anomalies between the study and control groups in total, females, and males (p > 0.05). A statistically significant difference was found between the distribution of developmental size, position, shape, and number anomalies between the study and control groups (p = 0.024). The most common anomaly in both groups was hypodontia (6.3% and 5.9%, respectively). There was a significant difference between the study and control groups in terms of subtypes of shape anomaly in all children and females (p = 0.045 and p = 0.05, respectively).

**Conclusions:**

While a significant difference was observed between the distributions of types of developmental dental anomalies between individuals with and without molar incisor hypomineralization, there was no difference in terms of the frequency of developmental dental anomalies.

## Background

Molar incisor hypomineralization (MIH), a type of developmental and qualitative enamel defect that affects at least one permanent first molar and, depending on its severity, also affects the permanent incisors, was first described two decades ago [1]. In the meta-analysis as a result of prevalence data obtained from 70 different studies, the global prevalence of MIH was reported to be 14.2% [2]. An average of 878 million people suffer from MIH, with more than 17.5 million new cases each year [3]. MIH, which is the most common developmental enamel defect from an epidemiological standpoint [4], affects nearly one out of every seven children, therefore, considering its global prevalence, it is important for public oral and dental health [5].

Although its etiology has not been clarified yet, it is known that some effects and changes on the organism before, during, and after birth may cause MIH. Etiological studies conducted to date have focused on maternal diseases, stress, medication, alcohol, and cigarette usage in the prenatal period; low birth weight, birth hypoxia, preterm birth, and other defects observed at birth; childhood diseases, exposure to environmental toxins, and long-term and frequent medication usage in the postnatal period [6–10]. It has also been shown that various genetic and epigenetic variants may be involved in the etiology of MIH [6, 11–14].

Developmental dental anomalies can manifest as shape, form, number, and structural anomalies in the dentition, depending on the abnormal conditions and interactions in the embryological development process [15, 16]. Although the etiology of developmental dental anomalies, as in MIH, remains largely uncertain, many studies have been conducted to evaluate genetic and environmental factors in the origin of these anomalies [17–20]. It has been reported that mutations in many gene families such as Msh homeobox 1 (MSX1), fibroblasts growth factor (FGF), paired box 9 (PAX9), ectodysplasin A (EDA), bone morphogenetic proteins (BMP), runt-related transcription factor 2 (RUNX2), adenomatous polyposis coli (APC), sonic hedgehog signaling molecule (SHH) may play a role in the origin of different developmental dental anomalies [21–24]. Brook [21, 25] noted that repeated signaling patterns over time during the sequential processes of initiation and morphogenesis are reflected not only in the tubercles of molars but also in the clinical presentation of the association of anomalies of number, size, and shape in the dentition. Consistent with the multilayered nature of the process, clinical outcome correlates with evidence of tissue changes and molecular genetic-epigenetic-environmental interactions [21]. Defects can occur when one or more components of teeth or dento-skeletal development are compromised during amelogenesis [26]. In addition, environmental effects, which are accepted as an essential factor in the etiology of both MIH and developmental dental anomalies, should not be ignored. Several gene families and mutations may play a role in the etiology of both MIH and developmental dental anomalies. Therefore, it was possible for various developmental dental anomalies to be observed in MIH and to affect dental development [27].

Given the knowledge that environmental and genetic/epigenetic effects play a common role in the etiology of both MIH and developmental dental anomalies, the aim of this study is to evaluate the presence of other developmental dental anomalies in children with and without MIH. The null hypothesis of the study is that there is no difference between the groups with and without MIH in terms of developmental dental anomalies.

## Methods

### Ethical approval and study population

The study protocol was assessed and approved by the Marmara University School of Dentistry Clinical Research Ethical Committee with approval number 2020 − 403. Written informed consent was obtained from the parents or legal guardians of all subjects in the study. The study was conducted in accordance with the principles of medical research involving human subjects stated in the Declaration of Helsinki. This cross-sectional and case-control study was conducted by analyzing digital panoramic radiographs of children admitted to pediatric dentistry clinics at the School of Dentistry, Marmara University, for routine dental care between February 2019 and August 2021. Children with MIH diagnosis in their electronic dental records were included in the study group, and children without MIH diagnosis were selected as the control group. Radiographs of 429 children with MIH (study group) were obtained with a nonrandom convenience sampling and 437 children without MIH (control group) obtained with random sampling were used to record developmental dental anomalies. For the control group, 437 radiographs were selected using the random.org website’s random integer generator tool among the children who met the inclusion criteria and did not present MIH.

### Sample size calculation

The population sample comprised all children aged 8–14 years with panoramic radiographs during the study period. The sample size was calculated based on Walshaw et al.’s study [28], which reported the prevalence of developmental dental anomalies in patients with MIH as 29%. Using the online software Sampsize (http://sampsize.sourceforge.net/iface/s3.html), the minimum sample to be included in the MIH group was 271, based on the following parameters: precision 5%, prevalence 29%, confidence interval 95%, and population 1869.

### Sampling criteria

The children included in the study, from whom the panoramic radiographs were obtained, were Turkish children living in Istanbul and neighboring provinces. Children undergoing routine dental treatments at the clinic, having panoramic radiographs, and examined by a single experienced and trained pediatric dentist (R.K.) in their dental records were enrolled in the study. Children with a diagnosis of MIH in their dental records were allocated to the MIH group, while those without MIH were allocated to the control group. The assessment of dental anomalies was conducted using panoramic radiographs. Teeth affected by MIH were evaluated according to the criteria accepted and approved by the European Academy of Paediatric Dentistry [29]. These criteria are as follows: no observable enamel defects; enamel defects that are not associated with MIH; whitish-creamy and/or yellowish-brownish demarcated opacities; post-eruptive enamel breakdown; atypical restorations; atypical caries; tooth loss because of MIH; and unscored situations [29]. It is known that the ideal age to diagnose MIH is 8 years old [30], whereas the maximum age for applying to the pediatric dentistry clinics at the School of Dentistry at Marmara University is 14. For these reasons, panoramic radiographs of children aged 8–14 years were included in the study. Patients without systemic disease who had digital panoramic radiography for their routine treatment, not specifically for the purposes of this study, were included in the study. Exclusion criteria were as follows: children under fixed orthodontic treatment or with a fixed space maintainer; children with a history of dental and/or craniofacial trauma, cleft lip and palate, and other craniofacial anomalies that may create developmental dental anomalies; children with other systemic developmental anomalies that may be associated with dental anomalies; children with tooth loss due to dental and/or craniofacial trauma or orthodontic reasons; and children whose radiographs were diagnostically unacceptable [28, 31, 32]. Third molars were not evaluated for the presence of developmental dental anomalies.

### Panoramic radiographs

Digital panoramic radiographs taken with the Morita device (VeraView IC5, J. Morita MFG. Corporation, Kyoto, Japan; kilovoltage peak 60–70, milliampere 7.5, time 8.8 s) at the oral and maxillofacial radiology clinics of the School of Dentistry, Marmara University, were used. A total of 445 radiographs for each group were evaluated by two pediatric dentists (R.K., N.T.) according to national guidelines published by the National Radiological Protection Board quality standards as follows: 1 = diagnostically excellent; 2 = diagnostically acceptable; and 3 = diagnostically unacceptable. These quality standards require at least 70% of all radiographs to be grade 1, less than 20% to be grade 2, and less than 10% to be grade 3 [33]. Radiographs graded as diagnostically unacceptable with a grade 3 were excluded from this study. Twenty-four of the 890 panoramic radiographs were excluded because they were Grade 3.

### Evaluation of developmental dental anomalies

Twelve different developmental dental anomalies were categorized into four types: size (microdontia, macrodontia); position (ectopic eruption of maxillary permanent first molars, infraocclusion of primary molars); shape (fusion, gemination, dilaceration, taurodontism, peg-shaped maxillary lateral incisors); and number (hypodontia, oligodontia, hyperdontia) anomalies [28, 31, 34, 35]. The following diagnostic criteria were used when detecting developmental dental anomalies:

Size anomalies:

- Macrodontia — a tooth with a wider mesiodistal width of the crown than usual relative to its contralateral homolog [16].
- Microdontia — a tooth with a narrower mesiodistal width of the crown than usual relative to its contralateral homolog [36].

Position anomalies:

- Ectopic eruption of maxillary permanent first molars — is a local eruption disturbance where the distal surface of the second primary molars blocks the permanent first molars, causing the permanent tooth to erupt to the occlusal plane and leading to pathological resorption of the roots of the second primary molars [37].
- Infraocclusion of primary molars — which is more common in primary molars, is a condition of tooth eruption in which the occlusal surface of the tooth is slightly depressed from the occlusal plane. If the distance to the occlusal plane is large, it can be seen that the adjacent teeth are inclined towards the infraocclusion tooth [38].

Shape anomalies:

- Fusion — The union of two separate tooth buds from enamel or dentin [16].
- Gemination — Incomplete formation of two teeth due to the division of a single tooth germ by invagination, resulting in an increase in the number of teeth in the dental arch [16].
- Dilaceration — is an abnormal angulation or bending of the long axes of the tooth crown and root [39].
- Taurodontism — Cases in which the tooth crown and/or pulp chamber grew vertically and the pulp chamber was observed in a rectangular configuration [40].
- Peg-shaped maxillary lateral incisor — maxillary lateral incisor whose mesio-distal width is narrower at the incisal than at the cervical [16].

Number anomalies:

- Hypodontia — Cases where there was no sign of crown calcification on the radiograph and no evidence of tooth loss attributable to trauma, caries, periodontal, or orthodontic causes in the child’s dental records [41].
- Oligodontia — Cases where the number of missing teeth is six or more (excluding third molars) [42].

- Hyperdontia — Cases in which teeth were present in addition to normal teeth [43].

### Statistical analyses

All panoramic radiographs were evaluated by two different and well-trained pediatric dentists (R.K., N.T.). Before the principal assessment, both examiners were trained by three different experienced investigators (B.Ş.Y., B.S., and B.K.) about MIH and other developmental dental anomalies. Two weeks after the initial evaluation, for intra- and inter-examiner agreement, randomly selected 50 panoramic X-rays from the study group and 50 from the control group were re-evaluated by both examiners. Intra- and inter-examiner reliability was assessed with Cohen’s Kappa test and assessed according to the categories suggested by Landis and Koch [44].

The mean and standard deviation were determined for the age variable. Descriptive statistics were presented as frequencies and percentages. Relationships between categorical variables were analyzed using multiple logistic regression. A binary logistic regression model was fitted for the children with developmental dental anomalies / without developmental dental anomalies as the dependent variable. This model included: the presence of MIH diagnosis and sexes. Since the presence of sex in the model was not significant, it was not included in the subsequent models. Data analysis was performed using SPSS (Statistical Package for the Social Sciences) version 26.0 software (The International Business Machines Corporation, Chicago, Illinois, United States of America), and p < 0.05 was considered statistically significant.

## Results

The intra-examiner Kappa value was 0.867 for examiner 1 (almost perfect) and 0.901 for examiner 2 (almost perfect), and the inter-examiner Kappa value was 0.845 (almost perfect).

Data were obtained from 866 patients, 429 with MIH (the study group) and 437 without MIH (the control group), who took panoramic radiographs while routine clinical care was ongoing. The mean age of the patients was 9.81 (standard deviation = 1.74). The percentage (88%) of diagnostically excellent radiographs (study group = 85.8%, control group = 90.2%) was greater than the National Radiation Protection Board’s recommendation (> 70%). The percentage (12%) of diagnostically acceptable radiographs (study group = 14.2%, control group = 9.8%) was lower than the recommended rate of 20%. 51.8% of the patients (n = 449) were female, and 48.2% were male (n = 417).

**Fig. 1:**
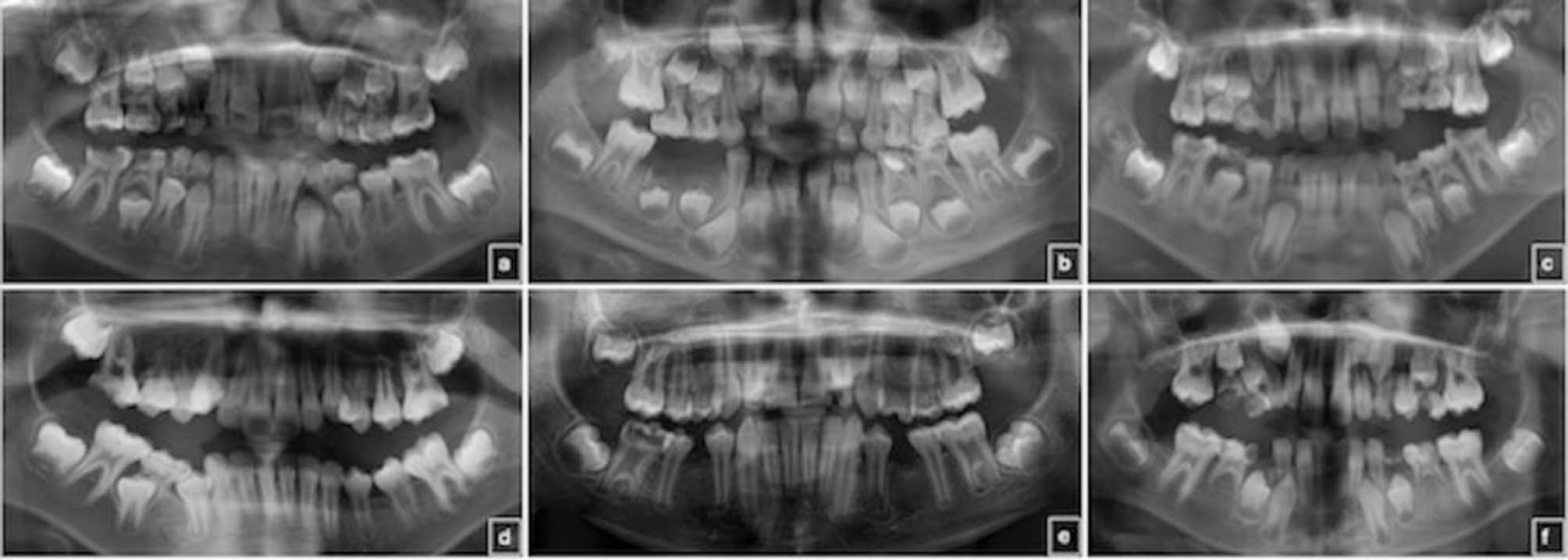
Representations from panoramic radiographs of children diagnosed with molar incisor hypomineralization and developmental dental anomalies. Macrodontia in maxillary central incisors is in **a**; ectopic eruption of maxillary permanent first molars is in **b**; infraocclusion of primary molars in left and right maxillary and left mandibular posterior regions is in **c**; dilaceration of the left mandibular permanent first molar root is in **d**; taurodontism in all permanent first molars is in **e**; and oligodontia is in **f**

In total, 12% (n = 102) of patients were found to have developmental dental anomalies. Examples of panoramic radiographs of children in the study group with developmental dental anomalies are shown in Fig. 1. Macrodontia in maxillary central incisors is in Fig. 1a; ectopic eruption of maxillary permanent first molars is in Fig. 1b; infraocclusion of primary molars in left and right maxillary and left mandibular posterior regions is in Fig. 1c; dilaceration of the left mandibular permanent first molar root is in Fig. 1d; taurodontism in all permanent first molars is in Fig. 1e; and oligodontia is in Fig. 1f. In nine patients, the same dental anomaly (such as symmetrical hypodontia or taurodontism, including all permanent first molars) was observed in more than one tooth. The most common teeth with developmental dental anomalies were the maxillary lateral incisors (n = 27), followed by mandibular premolars (n = 23). Hypodontia (n = 17), peg-shaped laterals (n = 4), hyperdontia (n = 3), macrodontia (n = 1), dilaceration (n = 1), and ectopic eruption (n = 1) were observed in the maxillary lateral incisors, while hypodontia (n = 21), dilaceration (n = 1), and ectopic eruption (n = 1) were observed in the mandibular premolars. The frequency of developmental dental anomalies in all children, females, and males, in the study and control groups is presented in Table 1. No significant difference was observed in the frequency of developmental dental anomalies between the study and control groups for all children, females, and males (p > 0.05) (Table 2). The frequency of developmental dental anomalies between females and males was also statistically insignificant (p = 0.275).

**Table 1:** The frequencies of developmental dental anomalies presented in total subjects, females, and males, in the study and control groups

	Variables	MIH n (%)	Control n (%)
Total	Children without developmental dental anomalies	374 (87.2)	390 (89.2)
	Children with developmental dental anomalies	55 (12.8)	47 (10.8)
Female	Children without developmental dental anomalies	208 (88.1)	193 (90.6)
	Children with developmental dental anomalies	28 (11.9)	20 (9.4)
Male	Children without developmental dental anomalies	166 (86)	197 (87.9)
	Children with developmental dental anomalies	27 (14)	27 (12.1)
n: number, MIH: Molar incisor hypomineralization			

**Table 2:** Factors influencing the presence of developmental dental anomalies by binary logistic regression analysis

		Odds Ratio	95% Confidence Interval	P^†^
Total subjects	**Males compared to females**	0.79	0.52–1.20	0.275
	**MIH compared to control**	0.81	0.53–1.22	0.313
Female	**MIH compared to control**	0.77	0.42–1.41	0.398
Male	**MIH compared to control**	0.84	0.48–1.49	0.557

MIH: Molar incisor hypomineralization, ^†^Binary logistic regression

A statistically significant difference was found between the distribution of developmental size, position, shape, and number anomalies between the study and control groups (p = 0.024) (Table 3). Macrodont teeth (n = 3), infraocclusion of primary molars (n = 2), and peg-shaped maxillary lateral incisors (n = 4) were detected only in the study group, while oligodontia (n = 2) was detected only in the control group. The most common developmental dental anomaly in both groups was hypodontia; the most common missing teeth were mandibular second premolars (n = 19), followed by maxillary lateral incisors (n = 14). When the types of developmental dental anomalies (position, shape, and number anomalies) were evaluated in the study and control groups, no significant difference was observed in total and between sexes (Table 4). In the comparison of position, shape, and number anomalies in the study group, no statistically significant difference was found according to the control group for all children, females, and males (p > 0.05) (Table 5). When subgroups of developmental dental anomalies were compared, a statistically significant difference was found between the study and control groups in terms of subtypes of shape anomalies in all children and females (p = 0.045 and p = 0.05, respectively) (Table 6). Microdont, fused, and geminated teeth were not observed in this study.

**Table 3:** Distribution of developmental dental anomalies in the study and control groups

Dental anomalies	MIH n (%)	Control n (%)	p^‡^
Macrodontia	3 (0.7)	0	**0.024**
Ectopic eruption of maxillary PFMs	6 (1.4)	6 (1.4)	
Infraocclusion of primary molars	2 (0.5)	0	
Dilaceration	2 (0.5)	3 (0.7)	
Taurodontism	6 (1.4)	2 (0.5)	
Peg-shaped maxillary lateral incisors	4 (0.9)	0	
Hypodontia	27 (6.3)	26 (5.9)	
Oligodontia	0	2 (0.5)	
Hyperdontia	5 (1.2)	8 (1.8)	
Total	55 (12.8)	47 (10.8)	

n: number, MIH: Molar incisor hypomineralization, PFM: Permanent first molar, ^‡^Multiple Univariate Logistic Regression, Bold font: p < 0.05

**Table 4:** Comparison of the types of developmental dental anomalies in the study and control groups in all subjects, females, and males

	Dental anomalies	MIH n (%)	Control n (%)	p^‡^
Total	Size anomalies	3 (5.5)	0	0.071
	Positional anomalies	8 (14.5)	6 (12.8)	
	Shape anomalies	12 (21.8)	5 (10.6)	
	Number anomalies	32 (58.2)	36 (76.6)	
	Total	55 (100)	47 (100)	
Female	Size anomalies	2 (7.1)	0	0.115
	Positional anomalies	3 (10.7)	5 (16.7)	
	Shape anomalies	8 (28.6)	2 (10)	
	Number anomalies	15 (53.6)	13 (65)	
	Total	28 (100)	20 (100)	
Male	Size anomalies	1 (3.7)	0	0.148
	Positional anomalies	5 (18.5)	1 (3.7)	
	Shape anomalies	4 (14.8)	3 (11.1)	
	Number anomalies	17 (63)	23 (85.2)	
	Total	27 (100)	27 (100)	

n: number, MIH: Molar incisor hypomineralization, ^‡^Multiple Univariate Logistic Regression

**Table 5:** Comparison of the types of developmental dental anomalies in the study and control groups in all subjects, females, and males, compared to children without anomalies

	Dental anomalies	MIH n (%)	Control n (%)	Odds Ratio	95% Confidence Interval	p^‡^
Total	Size anomalies	3 (5.5)	0	n.a.*		
	Positional anomalies	8 (14.5)	6 (12.8)	0.72	0.25–2.09	0.545
	Shape anomalies	12 (21.8)	5 (10.6)	0.40	0.14–1.15	0.088
	Number anomalies	32 (58.2)	36 (76.6)	1.08	0.66–1.77	0.765
	Total	55 (100)	47 (100)			
Female	Size anomalies	2 (7.1)	0	n.a.*		
	Positional anomalies	3 (10.7)	5 (16.7)	1.80	0.42–7.62	0.427
	Shape anomalies	8 (28.6)	2 (10)	0.27	0.06–1.29	0.100
	Number anomalies	15 (53.6)	13 (65)	0.93	0.43–2.01	0.862
	Total	28 (100)	20 (100)			
Male	Size anomalies	1 (3.7)	0	n.a.*		
	Positional anomalies	5 (18.5)	1 (3.7)	0.17	0.02–1.46	0.106
	Shape anomalies	4 (14.8)	3 (11.1)	0.63	0.14–2.86	0.552
	Number anomalies	17 (63)	23 (85.2)	1.14	0.59–2.20	0.687
	Total	27 (100)	27 (100)			

n: number, MIH: Molar incisor hypomineralization, ^‡^Multiple Logistic Regression, *not applicable due to small sample size

**Table 6:** Comparison of the subgroups of developmental dental anomalies in the study and control groups in all subjects, females, and males

Dental anomalies		MIH n (%)	Control n (%)	p^‡^
Positional anomalies				
Total	Ectopic eruption of maxillary PFMs	6 (75)	6 (100)	0.245
	Infraocclusion of primary molars	2 (25)	0	
Female	Ectopic eruption of maxillary PFMs	3 (100)	5 (100)	0.388
	Infraocclusion of primary molars	0	0	
Male	Ectopic eruption of maxillary PFMs	3 (60)	1 (100)	0.106
	Infraocclusion of primary molars	2 (40)	0	
Shape anomalies				
Total	Dilaceration	2 (16.7)	3 (60)	**0.045**
	Taurodontism	6 (50)	2 (40)	
	Peg-shaped maxillary lateral incisors	4 (33.3)	0	
Female	Dilaceration	2 (25)	2 (40)	**0.05**
	Taurodontism	4 (50)	0	
	Peg-shaped maxillary lateral incisors	2 (25)	0	
Male	Dilaceration	0	1 (33.3)	0.226
	Taurodontism	2 (50)	2 (66.7)	
	Peg-shaped maxillary lateral incisors	2 (50)	0	
Number anomalies				
Total	Hypodontia	27 (84.4)	28 (77.8)	0.718
	Hyperdontia	5 (15.6)	8 (22.2)	
Female	Hypodontia	14 (93.3)	11 (84.6)	0.752
	Hyperdontia	1 (6.7)	2 (15.4)	
Male	Hypodontia	13 (76.5)	17 (73.9)	0.865
	Hyperdontia	4 (23.5)	6 (26.1)	

n: number, MIH: Molar incisor hypomineralization, PFM: Permanent first molar, ^‡^Multiple Univariate Logistic Regression, Bold font: p < 0.05

## Discussion

In this study conducted on the idea that different dental anomalies originate from a common etiological factor, especially genetic factors, no relationship was found in terms of the frequencies of MIH and developmental dental anomalies. On the other hand, the difference in the distribution of developmental dental anomalies between children with and without MIH is statistically significant. The most common dental anomaly in both the study and control groups was hypodontia.

In a study conducted by Walshaw et al. [28], panoramic radiographs of 101 children aged 6–15 years diagnosed with MIH were evaluated for the presence of other developmental dental anomalies. In this study, the sample group, which did not include the control group, was made up of a small population. As the researchers suggested in their article, similar studies with a larger sample group are needed in a way that would include the control group. We planned the current study not only in line with the recommendations of Walshaw et al. [28], but also because the etiology of MIH and developmental dental anomalies is still not fully elucidated. Although the effect of different gene families on the etiology of MIH is still being investigated, the presence of systemic and environmental factors in the emergence of the disease remains unclear. Although studies to date have investigated the effects of different genes in MIH and developmental dental anomalies, it is possible that certain gene families contribute to the development of both conditions. Therefore, our study may actually form the basis for advanced genetic studies that will lead to the investigation of various gene families. To the best of our knowledge, this is the first study in the literature to investigate the relationship between the presence of MIH and developmental dental anomalies, while also including a control group.

In this study, there was no statistically significant difference in the evaluation of developmental dental anomalies on panoramic radiographs between children with and those without MIH. Although there is no other study in the literature that makes this evaluation in the presence of MIH, there are studies evaluating the presence of impacted canine teeth [45], cleft lip and palate [46, 47], velocardiofacial syndrome [32], nephrotic syndrome [35], and childhood cancers [31]. Nagpal et al. [45] reported that the developmental dental anomalies observed in the maxillary lateral incisors and taurodontism were significantly different between the groups in their evaluation on panoramic radiographs of a group of patients with and without impacted canines. In our study, although it was not statistically significant, the most common developmental dental anomalies were observed in the maxillary lateral teeth in both the study and control groups. Similarly, there was a quantitative increase in the occurrence of taurodontism in the MIH group. Investigating the presence and types of developmental dental anomalies in the cleft lip and palate, which are often of genetic origin, Camporesi et al. [46] reported that all anomalies differed statistically between the study and control groups, except for the second premolar agenesis. Furthermore, Germec Cakan et al. [47], who evaluated only the number and size anomalies in the maxilla, stated that a cleft palate significantly affects the lateral tooth agenesis on the affected side. Although no significant effect of velocardiofacial syndrome on developmental dental anomalies was reported [32], it was stated that hyperdontia, the presence of impacted teeth, number anomalies affecting more than one tooth, and shape anomalies of the incisors were significantly higher than their healthy peers in children with nephrotic syndrome [35]. On the other hand, Atif et al. [31] evaluated 120 childhood cancer survivors and 121 healthy peers without any other systemic disease in terms of developmental dental anomalies and developmental enamel defects. As a result of their study, it was reported that microdontia, abnormally shaped teeth, and developmental enamel defects were statistically different between the groups. The findings of this study support the idea that the presence of developmental enamel defects such as MIH may also make a difference in other developmental dental anomalies. Although it supports the hypothesis that forms the basis of our study, the findings of our study did not indicate a significant difference between the groups in terms of the presence and frequency of developmental dental anomalies.

As an important finding in the current study, a statistically significant difference was found in terms of the distribution of anomalies between the study and control groups. In addition, the difference in the distribution of shape anomalies in all subjects and females between the study and control groups was found to be statistically significant. Considering the genetic origin of developmental dental anomalies and the genetic and epigenetic studies on MIH, this finding is important. There are two studies [27, 48] in the literature examining tooth development on panoramic radiographs in patients with MIH. In the first one, tooth development was evaluated using the Demirjian method in panoramic radiographs of 105 children with severe MIH and 105 age- and sex-matched controls, and no significant difference was observed in terms of dental age and development between the two groups [48]. In the study of Sezer et al. [27], the dental development of 308 children with MIH and the same number of children who were matched for age and sex without MIH were evaluated with three different valid and reliable dental age estimation methods in panoramic radiographs. In conclusion, it was reported that MIH did not have a significant effect on dental age and development in the evaluation performed with the two most accurate methods. Although the findings of our study showed that there was no difference in the frequency of developmental dental anomalies in the study and control groups, there was a significant difference in the distribution of existing anomalies between the groups. This result is in line with the knowledge that different genetic effects may cause different anomalies and the findings of these studies.

In this study, the most common developmental dental anomaly observed in both the study and control groups was hypodontia. Although Walshaw et al. [28] stated that hypodontia is observed more frequently than other anomalies in the panoramic radiographs of children with MIH and should not be ignored, we think that hypodontia can actually be observed independently of MIH. This is also demonstrated by the statistically non-significant difference between the study and control groups. It is known that the global prevalence of hypodontia is between 2.3% and 10% [49, 50]. Considering that genetic factors can produce a variety of results in different birth years and cohorts together with environmental factors, the rates determined in non-syndromic hypodontia prevalence studies conducted in Türkiye in various years and geographical regions [6.2% (51), 6.7% (52), and 4.3% (53)] are consistent with the results of our study. Although studies have focused on the Msh homeobox 1 (MSX1) and paired box 9 (PAX9) genes in the etiology of non-syndromic hypodontia [49, 54], it is believed that mutations in various gene families may contribute to this condition [49]. Although there was no statistically significant difference in the presence of hypodontia between the groups with and without MIH in our study, we think that there is a need for different epigenetic and genetic studies that can be conducted on this subject.

Regardless of the presence of MIH, the prevalence of developmental dental anomalies in the entire sample group was determined to be 12%. While the prevalence of developmental dental anomalies was reported as 36% in the study of Sella Tunis et al. [55], in which they examined the data of a sample group of 2897 Caucasian individuals, this rate was found to be 20.9% in the study of Lagana et al. [42], in which they examined the data of 5005 samples. In their study on a group of Albanian orthodontic samples, Vinjolli et al. [56] reported the prevalence of at least one dental anomaly as 24.4%, while it was stated that more than one dental anomaly was seen in 4.6% of the cases. In a prevalence study conducted on a group of Nigerian children in primary and mixed dentition, it was reported that 26.6% of 1026 children had dental anomalies [16]. On the other hand, in another study conducted in Nigeria, it was stated that dental anomalies were clinically observed in only 4.2% of 1565 children between the ages of 12 and 15 [57]. In a study evaluating the frequency of developmental dental anomalies in the permanent dentition of orthodontic patients at the State University of New York, the prevalence was reported to be 20.4% [58], while in another study conducted on panoramic radiographs of 512 children between the ages of 6 and 12, 61.3% of the children had at least one dental anomaly reported [59]. The prevalence of developmental dental anomalies has a wide range, as they may have different etiological origins and be under the influence of different genetic factors. Furthermore, the fact that the prevalence varies even in different birth cohorts with different racial and environmental influences makes it difficult to establish a generalizable prevalence. While the prevalence observed in the entire sample group in our study is higher than the rates found in some studies, it is lower than the results of some studies.

Although there was no statistically significant difference between the study and control groups in terms of the presence and frequency of developmental dental anomalies, the hypothesis of our study was partially rejected since a significant difference was observed in the distribution of observed developmental dental anomalies. There are some limitations of our study. MIH is a multifactorial condition affected by different etiological factors. This situation varies according to environmental factors, year of birth, and even geographical region [27]. Therefore, the fact that only one geographic region and one age range were evaluated in our study limits the generalizability of the results. In addition, another limitation of the study is the evaluation of developmental dental anomalies with panoramic radiographs. Magnification and distortion in panoramic radiographs can lead to incorrect diagnosis of anomalies such as macrodontia and microdontia. However, to minimize this limitation, only diagnostically excellent radiographs were included in this study. On the other hand, the presence of a control group, a relatively large sample size, and the existence of intra- and inter-examiner agreement are the main strengths of our study.

## Conclusion

In conclusion, there is no significant difference in the frequency of developmental dental anomalies in children with molar incisor hypomineralization when compared to children who do not have molar incisor hypomineralization. The distribution of the observed developmental dental anomalies varies significantly between individuals with and without molar incisor hypomineralization. In addition, the most common developmental dental anomaly, independent of the presence of molar incisor hypomineralization, is hypodontia. Considering the increasing regional and global prevalence of molar incisor hypomineralization, more studies are needed in different populations, birth cohorts, in different geographic regions, with larger sample sizes, and evaluating through intraoral examinations in addition to radiographs.

## Data Availability

The datasets used and analyzed during the current study available from the corresponding author on reasonable request.
